# Surgical ward rounds in England: a trainee-led multi-centre study of current practice

**DOI:** 10.1186/1754-9493-8-11

**Published:** 2014-02-28

**Authors:** Ceri Rowlands, Shelly N Griffiths, Natalie S Blencowe, Alexander Brown, Andrew Hollowood, Steve T Hornby, Sarah K Richards, Jennifer Smith, Sean Strong

**Affiliations:** 1 Division of Surgery, Head and Neck, University Hospitals Bristol NHS Foundation Trust, Bristol BS2 8HW, England; 2 Torbay Hospital, Torquay, Devon TQ2 7AA, England; 3 Centre for Surgical Research, School of Social & Community Medicine, University of Bristol, Canynge Hall, Bristol BS8 2PS, England; 4 Musgrove Park Hospital, TauntonSomerset TA1 5DA, UK; 5 Royal Devon and Exeter Hospital, Barrack Road, Exeter EX2 5DW, England; 6 Royal United Hospital Bath NHS Trust, Combe Park, Bath BA1 3NG, England; 7 Royal Preston Hospital, Preston PR2 9HTLancashire, England

**Keywords:** Health manpower, Ward rounds, Seven day working, Consultant

## Abstract

**Background:**

Recent guidance advocates daily consultant-led ward rounds, conducted in the morning with the presence of senior nursing staff and minimising patients on outlying wards. These recommendations aim to improve patient management through timely investigations, treatment and discharge. This study sought to evaluate the current surgical ward round practices in England.

**Methods:**

Information regarding timing and staffing levels of surgical ward rounds was collected prospectively over a one-week period. The location of each patient was also documented. Two surgical trainee research collaboratives coordinated data collection from 19 hospitals and 13 surgical subspecialties.

**Results:**

Data from 471 ward rounds involving 5622 patient encounters was obtained. 367 (77.9%) ward rounds commenced before 9am. Of 422 weekday rounds, 190 (45%) were consultant-led compared with 33 of the 49 (67%) weekend rounds. 2474 (44%) patients were seen with a nurse present. 1518 patients (27%) were classified as outliers, with 361 ward rounds (67%) reporting at least one outlying patient.

**Conclusion:**

Recommendations for daily consultant-led multi disciplinary ward rounds are poorly implemented in surgical practice, and patients continue to be managed on outlying wards. Although strategies may be employed to improve nursing attendance on ward rounds, substantial changes to workforce planning would be required to deliver daily consultant-led care. An increasing political focus on patient outcomes at weekends may prompt changes in these areas.

## Introduction

Ward rounds (WRs) represent a complex interaction between clinical staff and patients and are crucial to providing safe, high-quality care in a timely and efficient manner. They allow opportunities to review diagnoses in light of clinical findings and investigations, formulate on-going management or discharge plans and facilitate information sharing between patients, relatives, and healthcare professionals whilst also playing an important role in training [1,2]. Despite these accepted merits, it has been suggested that they remain a much-neglected part of inpatient care [1,3].

Recently, best practice guidelines for this important clinical activity have been published [1,2]. These include ensuring the presence of a senior nurse during every bedside review and that rounds should take place early in the day to facilitate timely completion of tasks, such as requesting investigations and discharging patients. The importance of senior leadership on WRs has also been highlighted, recommending that a consultant should review patients at least once, every 24 hours [2]. This issue may be of particular relevance to surgical specialties, as a recently well-publicised study has suggested that outcomes amongst patients admitted during weekends, or undergoing elective procedures towards the end of the week, may be less favourable [4]. One proposed cause for this ‘weekend effect’ is the perceived lack of consultant input into patient management outside of routine working hours. Lastly, it has been proposed that patients should be nursed on appropriate speciality wards, rather than outlier beds, in order to improve care and aid early identification of problems and complications [1]. In light of the aforementioned guidelines and increasing interest in the provision of routine and unscheduled surgical care, we aimed to assess current surgical ward round practices in England.

## Methods

Hospitals were recruited using two trainee-led surgical research collaboratives in the South West and North West of England. Research collaboratives are networks of trainees which aim to promote collaboration in surgery in order to maximise the quality and applicability of research and audit studies [5]. Information regarding surgical WRs across multiple subspecialties was collected prospectively over a one-week period in February 2013.

The total number of WRs and individual patient encounters was recorded. WRs were classified as occurring on a weekday (Monday – Friday) or weekend (Saturday and Sunday) and by type, using an *a priori* categorisation system (Table 1). Data was recorded regarding: (i) start time of WR, (ii) seniority of staff leading WR, (iii) nursing presence at each patient encounter, and (iv) number of outlying patients. Seniority of staff leading the WRs was categorised as consultant or non-consultant. Non-consultant WRs were further classified as staff grade, clinical fellow, specialist trainee (ST3-ST8), core trainee (CT1-3) and foundation year (FY) doctors. The number of outlying patients, defined as any patient under the care of one specialty on the base ward of another specialty, was also recorded.

**Table 1:** Definitions of ward round categories

**Ward round type**	**Definition**
**Acute admissions**	Review of emergency admissions during the course of a 24 hour take period, often undertaken in the evening, distinct from the post-take ward round.
**Post-take**	Formal post-take round of emergency admissions, following completion of a 24 hour take period
**Post-operative**	Review of patients in the immediate post-operative period, often on an ad-hoc basis at the end of an operating list
**Daily working round**	Daily ward round of current inpatients under the care of a surgical firm
**Other**	Any round taking place not defined within a preceding category

## Results

Sixty-two surgical firms from nineteen hospitals participated in the study. A total of 471 individual WRs were recorded, of which 422 (89.6%) were carried out on weekdays. Collectively, these encompassed 5622 individual patient encounters, with 4915 from Monday to Friday and 707 on the weekend. The mean number of ward rounds was higher on weekdays than weekends (84.4 and 24.5, respectively) and similarly the mean number of patient encounters was higher on weekdays (983 and 353.5, respectively).

### Timing of WR

367 WRs (77.9%) began before 9am, and by 11am 90.4% had commenced (n = 426). Details of all WR start times are listed in Table 2.

**Table 2:** Ward round start times

**Start time**	**Number of ward rounds**
	**n = 471 (%)**
<0900	367 (77.9)
0900 – 0959	49 (10.3)
1000 – 1059	10 (2.1)
>1100	29 (6.2)
Not recorded	16 (3.4)

### Seniority of staff leading the ward round

Consultants led a total of 223 (47.3%) WRs. 190 of the 422 weekday rounds (45.0%) and 2126 of the 4915 patient encounters (43.2%) were led by consultants, compared with 33 of the 49 WRs (67.0%) and 550 of the 707 (77.8%) patient encounters during weekends (Table 3).

**Table 3:** Weekday and weekend ward rounds according to grade

**Grade of doctor leading ward round**		**Number of weekday rounds n = 422 (%)**	**Number of weekend rounds n = 49 (%)**
**Consultant**		190 (45.0)	33 (67.3)
**Non-consultant**	Staff grade	15 (3.6)	3 (6.1)
	Clinical fellow	13 (3.1)	0 (0.0)
	ST3+	135 (32.0)	7 (14.3)
	CT1-3	47 (11.1)	2 (4.1)
	FY1-2	13 (3.1)	2 (4.1)
**Unknown**		9 (2.1)	2 (4.1)

(ST3+ = Specialist Trainee, CT1-3 = Core Trainee, FY1-2 = Foundation Year Doctor).

### Nursing presence

2474 of the 5622 patient encounters (44.0%) occurred in conjunction with a member of nursing staff. Nursing presence was similar for consultant-led and specialty trainee-led patient encounters (1335/2676, 49.9% and 836/1837, 45.5%, respectively) compared to 5.0% (30/606) of those led by core trainees (Table 4). Nursing presence was also similar during weekdays (2169/4915, 44.1%) and at weekends (305/707, 43.1%).

**Table 4:** Nursing presence on ward rounds

**Grade of doctor leading patient encounter**		**Number of weekday encounters n = 4915**	**Number of weekday encounters with nurse present n = 2169 (%)**	**Number of weekend encounters n = 707**	**Number of weekend encounters with nurse present n = 305 (%)**
**Consultant**		2126	1106 (52.0)	550	229 (41.6)
**Non-consultant**	Staff grade	151	104 (68.9)	42	36 (85.7)
	Clinical fellow	86	59 (68.6)	15	10 (66.7)
	ST3+	1781	806 (43.6)	56	30 (53.6)
	CT1-3	572	30 (5.2)	34	0 (0)
	FY1-2	185	58 (36.3)	4	0
**Unknown**		14	6 (42.9)	6	0

(ST3+ = Specialist Trainee, CT1-3 = Core Trainee, FY1-2 = Foundation Year Doctor).

### Outliers

1274 (22.7%) of the 5622 patient encounters were undertaken on outlying wards. The proportion of outlying patients varied between subspecialties (3.3%, neurosurgery to 33.0%, transplant surgery) (Table 5).

**Table 5:** Outliers by surgical subspecialty

**Surgical specialty**	**Total number of patient encounters**	**Number of patients on outlying wards (%)**
**Orthopaedic surgery**	974	245 (25.2)
**Lower GI surgery**	882	202 (22.9)
**Urology**	584	173 (29.6)
**Upper GI surgery**	574	97 (16.9)
**Vascular surgery**	433	79 (18.2)
**Breast surgery**	227	67 (29.5)
**‘General surgery’**	1111	259 (23.3)
**Ear, nose and throat surgery**	206	62 (30.0)
**Obstetrics and gynaecology**	131	14 (10.7)
**Paediatric surgery**	100	16 (16.0)
**Plastic surgery**	107	8 (7.5)
**Transplant surgery**	97	32 (33.0)
**Oral and maxillofacial surgery**	16	3 (18.8)
**Thoracic surgery**	90	14 (15.6)
**Neurosurgery**	90	3 (3.3)

## Discussion

This national multi-centre study included 5622 patient encounters from 471 surgical WRs. The majority of WRs occurred before 0900 (77.9%) and many patients (77.3%) were nursed on appropriate wards. However, less than 50% of WRs were led by consultants and 56% of patient encounters occurred without a nurse at the bedside. Although weekend WRs were more likely to be consultant-led, the mean number of WRs and patient encounters per day fell dramatically over the weekend period and the level of nursing presence on WRs was lacking during weekdays (44.1%) and on weekends (43.1%). We have found that compliance with recent recommendations for WR practice is therefore poor, and warrants further attention if standards are to be improved.

The importance of hospital WRs has been highlighted through a number of publications investigating their clinical benefits [6-10]. Following the introduction of twice-daily consultant WRs in a large UK medical centre, average length of stay fell from 10.4 to 5.3 days (p < 0.01) without increasing readmissions [6]. Consultant-led surgical post-take WRs can also change initial admitting diagnoses in up to 27% of cases [7]. In another study, the use of a multidisciplinary ward round increased their educational benefit whilst also reducing length of hospital stay [8] and similarly, the presence of registered nurses has been shown to reduce adverse events and mortality [9].

To our knowledge, this is the first national multi-centre study to prospectively examine surgical ward round practice in the UK; nevertheless, it has several limitations. Although the number of WRs and patient encounters fell dramatically over the weekend period, we were unable to identify the exact number of inpatients who were not reviewed, or who were discharged, during every 24 hour period. The number of patients discharged may, therefore, influence the number of weekend patient encounters, and it is also possible that ad-hoc ward rounds conducted by consultants without junior staff may not have been recorded. It is unlikely, however, that these factors account for the vast differences observed between weekdays and weekends. Furthermore, a single seven day snapshot may not accurately reflect normal practice, although a wide cross-section of subspecialties was surveyed across three teaching deaneries and change-over weeks and school holidays were deliberately avoided. Lastly, we acknowledge that the number of included WRs from some sub-specialities is low, meaning that variations in practice cannot accurately be assessed.

Our study highlights many challenges for the future, if the clinical standards regarding WRs are to be achieved. The current drive within the NHS is towards ’24-7′ consultant-delivered care [10], with patients nursed in appropriate ward settings [1] in order to reduce avoidable hospital deaths [11]. Increasing the proportion of patients reviewed at weekends represents a potential area for improvement, particularly given the purported increased mortality during this time period [3] and local strategies could be employed to improve nursing attendance at bedside reviews.

Scrutinising the performance of surgical units through the recent move to publish surgeons’ outcome data [12,13] represents a culture change throughout the NHS. Seven day working strategies in surgery are seemingly inevitable [14], but the entire hospital and allied services may also require full staffing seven days a week in order to achieve this. If WRs are to become consultant-led seven days a week, alterations to their working patterns will be required. For example, it is estimated that a consultant ward round to review 30 inpatients would take 6 hours [15], which would necessitate removal of the equivalent time from existing weekday clinical, managerial or teaching commitments. These represent substantial changes and confer cost implications as well as affecting the provision of specialised elective services and potentially, the quality of surgical training in the UK.

## Appendix 1

### Trainee collaborators; Severn and Peninsula Audit and Research Collaborative for Surgeons

V Aggarwal; V Agosti; F Ali; A Ashman; J Bagenal; K Ball; A Barrie; E Binns; H Blades; A Brennan; T Brimecombe; O Burdall; CVE Carpenter; A Chambers; A Chambers; T Chambers; GS Chauhan; V Chouhan; H Collins; J Collins; D Constantin; L Corbett; M Crockett; D Dass; N Daulatzai; V Donkin; H Donnelly; K Doughty; L Dwon; A Easterbrook; D Eden; H Farnsworth; A Gamper; M Ghisel; A Greenwood; K Hanks; T Hardy; M Halls; R Hinton; C Hockin; E Hodgson; C Honeyman; Y Hughes; A Jacob; J Jackson; L Jay; M Jones; D Kanakopoulos; S Kalyanapu; I Kear; F Khan; H Knight; N Lemay; S Linley; S Linthwaite; S Logarajah; N Lyn-White; M Marshall; P McElnay; A Moutsoudis; A Nagvi; R O'Byrne; O Old; S Olsen; E Orr; O Pearce; V Pegna; H Pidduck; E Platt; W Pollitt; J Potts; K Price; L Regan; A Raymond; D Urriza Rodriguez; A Ross; K Sahnan; L Salimin; E Saxby; S Scholes; G Silk; H Stark; H Stephenson; B Soukup; C Stockdale; J Sykes; M Taylor; A Toonah; H Travers; M Tulbure; H Tustin; D Twelves; D Teichmann, E Upchurch; M Vannahme; M Vipond; C Wallengren; T Walker; S Walter; J Warbrick-Smith; B Warwick; B Wasunna; T Wing; J Wolf; C Worall; **North West Research Collaborative;** C Goatman; L Patel; R Lamb; J Littlechild; I Maitra; M Williams; L Olson; S Hassan; H Collier; M Hussain.

## Appendix 2

### Consultant collaborators

S Dwerryhouse; P Eyers; N Gallegos; H Gilbert; S Higgs; K McCarthy; J Mutimer; WD Neary.

## Competing interests

The authors declare that they have no competing interests.

## Authors’ contributions

CR and SG were involved in study conception and design, were regional co-ordinators for data collection in Severn and Peninsula deaneries respectively, performed data analysis and manuscript drafting and editing. NSB, AB, AH, STH, SKR, SS were all involved in study conception and design and reviewing and editing of the manuscript. JS was regional co-ordinator for data collection in Northwest deanery and was involved in the reviewing and editing the manuscript. Severn and Peninsula Audit and research Collaborative for Surgeons and Northwest Research Collaborative would like to kindly thank all persons named in Appendix 1 and Appendix 2 for their important role in coordinating data collection in their respective local centres. All authors read and approved the final manuscript.
